# Transcriptional regulation of Notch1 by nuclear factor-κB during T cell activation

**DOI:** 10.1038/s41598-022-26674-1

**Published:** 2023-01-02

**Authors:** Jeong-Ryul Hwang, Donghwan Kim, Jung-Ah Kang, Sang-Heon Park, Sung-Gyoo Park

**Affiliations:** 1 Institute of Pharmaceutical Sciences, College of Pharmacy, Seoul National University, Seoul, Republic of Korea; 2 School of Life Sciences, Gwangju Institute of Science and Technology (GIST), Gwangju, Republic of Korea; 3 Bio-Nanotechnology Research Center, Korea Research Institute of Bioscience and Biotechnology, Daejeon, Republic of Korea

**Keywords:** CD4-positive T cells, Immunology, Molecular biology

## Abstract

Notch1 plays important roles in T cell development and is highly expressed in activated CD4^+^ T cells. However, the underlying mechanism of *Notch1* transcription in T cells has not been fully characterized. Therefore, we aimed to determine how Notch1 expression is regulated during the activation of CD4^+^ T cells. Both the surface expression and mRNA transcription of Notch1 were significantly higher in activated CD4^+^ T cells, but the inhibition of phosphatidylinositol 3-kinase (PI3K) by LY294002 or deletion of the *Pdk1* gene impaired this upregulation of Notch1. Interrogation of the *Notch1* promoter region using serially deleted *Notch1* promoter reporters revealed that the − 300 to − 270 region is crucial for its transcription in activated T cells. In addition, we found that nuclear factor (NF)-κB subunits containing RelA bind directly to this promoter region, thereby upregulating transcription. In addition, inhibition of NF-κB by SN50 impaired upregulation of Notch1 surface protein and mRNA in activated CD4^+^ T cells. Thus, we provide evidence that *Notch1* transcription in activated CD4^+^ T cells is upregulated via the PI3K-PDK1-NF-κB signaling pathway.

## Introduction

The Notch family comprises four conserved transmembrane receptors (Notch 1–4) that contain an ectodomain with epidermal growth factor-like repeats; an intracellular domain containing a regulation of amino acid metabolism (RAM) 23 domain, ankyrin repeats, and a proline, glutamate, serine, threonine-rich (PEST) domain^1^. These are single-pass transmembrane heterodimeric receptors, in which the N-terminal extracellular subunit and the C-terminal intracellular subunit, which includes a transmembrane domain, are noncovalently associated^2^. Notch signaling is triggered by the binding of ligands, which include Jagged 1, Jagged 2, Delta-like 1, Delta-like 3, and Delta-like 4^3–5^. When a ligand binds to Notch1, the extracellular domain of Notch1 is cleaved by the ADAM10 metalloprotease, and subsequent proteolytic cleavage occurs in the intracellular domain as a result of activity of the γ-secretase complex^6–8^. The released Notch1 intracellular domain then translocates to the nucleus, where it regulates transcription^6^. Specifically, the cleaved Notch1 intracellular domain interacts with a recombination signal binding protein for immunoglobulin kappa J region (RBP-Jκ), thereby converting it from a transcriptional repressor to an activator, which results in target gene expression^9^.

Notch1 plays a key role in T cell development and lineage commitment. Hematopoietic stem cell-derived progenitors from the bone marrow have the potential to become either T or B cells^10^. Studies of the overexpression of the active form and knockout of Notch1 have shown that Notch1 signaling affects whether progenitor cells are directed down the T or B cell lineage, promoting T cell, rather than B cell, differentiation^11,12^. After commitment to the T cell lineage, the T cell progenitor cells differentiate into αβ or γδ T cells. Previous studies using mouse model systems suggest that a reduction in Notch1 signaling increases differentiation of γδ T cells at the expense of αβ T cells^13,14^. However, the opposite effect was found in human cells^15^. In addition, Notch signaling regulates various aspects of T cell function, including their activation, differentiation, expansion, and cytokine production^9^. Furthermore, recent studies of T cell acute lymphoblastic leukemia (T-ALL) have revealed that an activating mutation in Notch1 commonly causes leukemia in cooperation with other oncogenic mutations^16^. Therefore, Notch1 has emerged as an important therapeutic target for T-ALL.

Despite the importance of Notch1 in T cells, it remains unclear how *Notch1* expression is regulated in this cell type. There have been some previous studies of the transcriptional regulation of *Notch1* in non-T cells. p53 is a well-known transcriptional regulator of *Notch1* in keratinocytes, in which it activates transcription by directly binding to the promoter region^17,18^. Kruppel-like factor (Klf) 4 and Sp3 are other transcriptional regulators of *Notch1* in keratinocytes, in which they bind to the *Notch1* promoter, causing a reduction in gene expression^19^. Recent studies show that distal-less homeobox (DLX) 5 binds directly to the *Notch1* promoter, which triggers upregulation of gene transcription in osteosarcoma cells, and that SIRT6 epigenetically regulates *Notch1* transcription via DNA methyltransferase (DNMT) 1 in adipose-derived stem cells^20,21^. However, their relevance for *Notch1* transcription in T cells has not been evaluated. Therefore, we aimed to determine how Notch1 expression is regulated in T cells by attempting to identify the most important promoter region for *Notch1* transcription and the key transcriptional regulators. To this end, we used serial deletions of the *Notch1* promoter region and found that the − 300 to − 270 region is critical for the transcription of the gene in CD4^+^ T cells. In addition, we found that the RelA-containing nuclear factor (NF)-κB complex proteins directly bind to this region to positively regulate transcription.

## Results

### Notch1 expression is induced by phosphoinositide 3-phosphate (PI3K)-phosphoinositide-dependent kinase-1 (PDK1) signaling

**Figure 1:**
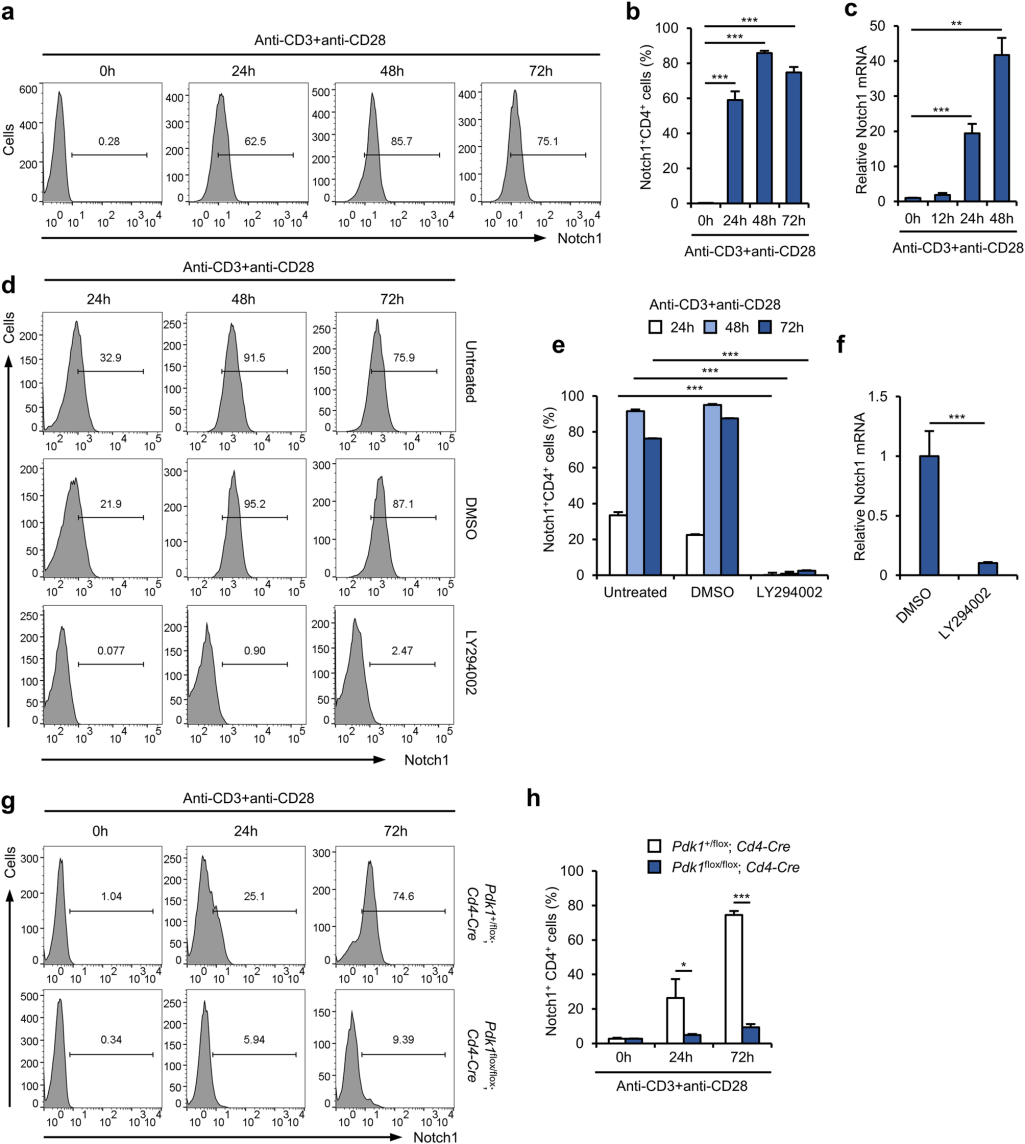
Notch1 expression is upregulated in activated primary CD4^+^ T cells via the PI3K-PDK1 pathway. (**a, b**) Flow cytometry for surface Notch1 expression after cellular activation using anti-CD3 and anti-CD28 antibodies for the indicated times. (**c**) Quantitative real-time PCR for *Notch1* mRNA expression after cellular activation. (**d, e**) Flow cytometry for surface Notch1 expression after treatment with the PI3K inhibitor LY294002. (**f**) Quantitative real-time PCR to detect *Notch1* mRNA after cells were activated by anti-CD3 and anti-CD28 antibodies for 48 h in the presence of LY294002. (**g, h**) Flow cytometry for surface Notch1 expression in PDK1-deficient CD4^+^ T cells. Data are mean ± SD. *P ≤ 0.05, **P ≤ 0.01, and ***P ≤ 0.001 (Student’s *t*-test).

We first determined whether Notch1 expression is regulated during T cell activation. Negatively isolated primary CD4^+^ T cells were activated using anti-CD3 and anti-CD28 antibodies, and flow cytometric analysis showed that the surface expression of Notch1 was much higher after activation (Fig. 1a, b). We next determined whether the high Notch1 surface expression was the result of upregulation of *Notch1* transcription, and found that *Notch1* mRNA expression increased in a time-dependent manner after activation using anti-CD3 and anti-CD28 antibodies (Fig. 1c). These findings imply that the high surface expression of Notch1 is principally the result of the transcriptional activation of *Notch1*. Interestingly, the activation-induced Notch1 expression was abolished by treatment with LY294002, a PI3K inhibitor. Over half of the activated CD4^+^ T cells showed an increase in surface Notch1 expression after 72 h, but almost none of the LY294002-treated CD4^+^ T cells upregulated Notch1 (Fig. 1d, e), which implies that the PI3K pathway plays a critical role in Notch1 expression. In addition, expression of Notch1 mRNA levels fell markedly in activated CD4^+^ T cells treated with LY294002 (Fig. 1f). We also checked whether reduced expression of Notch1 surface protein and mRNA is due to destabilization of Notch1 mRNA after treatment with LY294002; however, LY294002 treatment did not accelerate decay of Notch1 mRNA (Supplementary Fig. 1). Because PDK1 is a principal downstream effector of PI3K, we next measured Notch1 expression in primary CD4^+^ T cells isolated from mice with CD4^+^ T cell-specific deletion of PDK1 that were activated using anti-CD3 and anti-CD28 antibodies. We found that upregulation of Notch1 expression was impaired markedly in *Pdk1*-deficient CD4^+^ T cells (Fig. 1g, h). Thus, PI3K-PDK1 signaling increases Notch1 expression through a robust induction of transcription in activated CD4^+^ T cells.

### The − 300 to − 270 region of Notch1 is crucial for its transcription

**Figure 2:**
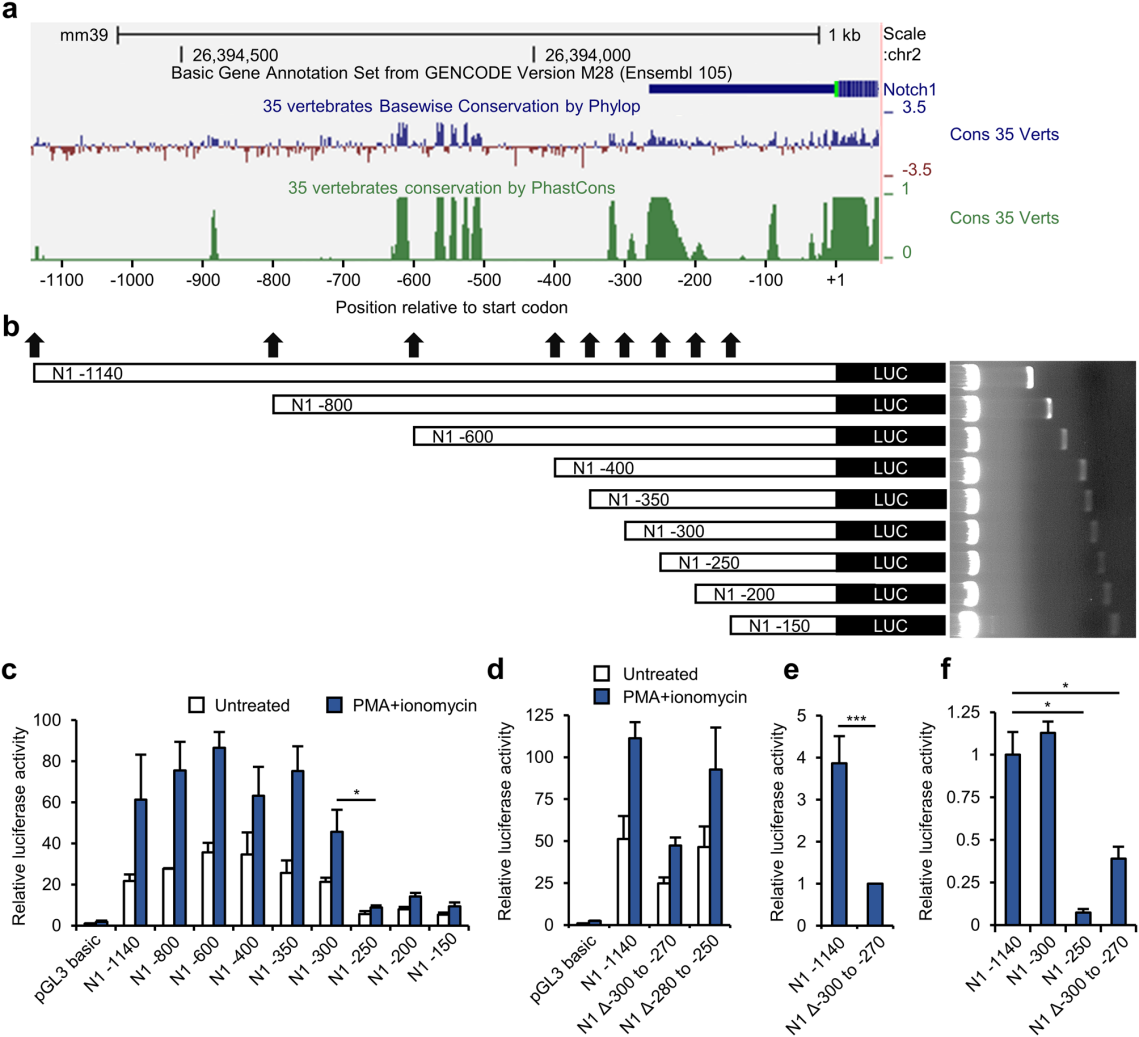
Identification of the key regulatory region of the putative *Notch1* promoter. (**a**) Conservation map of the mouse *Notch1* promoter region. (**b**) Schematic representation of the serially deleted *Notch1* promoter vector constructs. (**c–e**) Luciferase reporter assays performed in activated Jurkat T cells for identification of the key regulatory region using (**c**) transfection with truncated *Notch1* promoter reporter vectors, (**d**) transfection with internally deleted *Notch1* promoter reporter vectors, and (**e**) activation with anti-CD3 and anti-CD28 antibodies. (**f**) Luciferase reporter assay performed in activated CD4^+^ T cells transfected with *Notch1* promoter reporter vectors selected during steps **c–e**. Data are mean ± SD. *P ≤ 0.05, **P ≤ 0.01, and ***P ≤ 0.001 (Student’s *t*-test).

The upstream region (up to − 400 base pairs) from the start codon of *Notch1* is highly conserved among vertebrates; this conserved region continues intermittently until − 1000 base pairs (Fig. 2a). To identify the essential *cis* element for Notch1 transcriptional regulation, sections of the *Notch1* promoter region (+ 1 to − 1140) were serially deleted, and the deleted fragments were cloned into the pGL3 basic reporter plasmid (Fig. 2b). These vectors were transfected into Jurkat T cells, the cells were activated using PMA/ionomycin, and a luciferase reporter assay was performed. This showed a significant decrease in luciferase activity when the *Notch1* − 300 to − 250 region was deleted (Fig. 2c). To interrogate the *Notch1* − 300 to − 250 region in more detail, the *Notch1* − 300 to − 270 (N1 Δ − 300 to − 270) and − 280 to − 250 (N1 Δ − 280 to − 250) regions were deleted from the longest vector construct (N1 − 1140), and Jurkat T cells were transfected with each vector and stimulated with PMA/ionomycin. The N1 Δ − 300 to − 270-transfected cells exhibited a significant decrease in luciferase activity (Fig. 2d), whereas deletion of the *Notch1* − 280 to − 250 region did not substantially affect the luciferase activity (Fig. 2d). To evaluate the importance of the identified *cis* element for TCR/CD28-mediated activation, Jurkat T cells were transfected with N1 − 1140 or N1 Δ − 300 to − 270 and stimulated using anti-CD3 and anti-CD28 antibodies. Deletion of the *Notch1* − 300 to − 270 region considerably reduced the luciferase activity in cells stimulated using anti-CD3 and anti-CD28 antibodies (Fig. 2e). On the basis of these findings, we further interrogated the importance of the *Notch1* − 300 to − 270 region in primary CD4^+^ T cells. The luciferase reporter vectors were transfected into mouse CD4^+^ T cells by electroporation, and the cells were cocultured with antigen-presenting cells in the presence of soluble anti-CD3 and anti-CD28 antibodies. The luciferase activity of the cells was much lower when they were transfected with N1 − 250 or N1 Δ − 300 to − 270 (Fig. 2f). These findings suggest that the *Notch1* − 300 to − 270 region is important for the transcription of *Notch1* in primary mouse CD4^+^ T cells, as well as in Jurkat cells.

### NF-κB directly binds to the Notch1 − 300 to − 270 region

**Figure 3:**
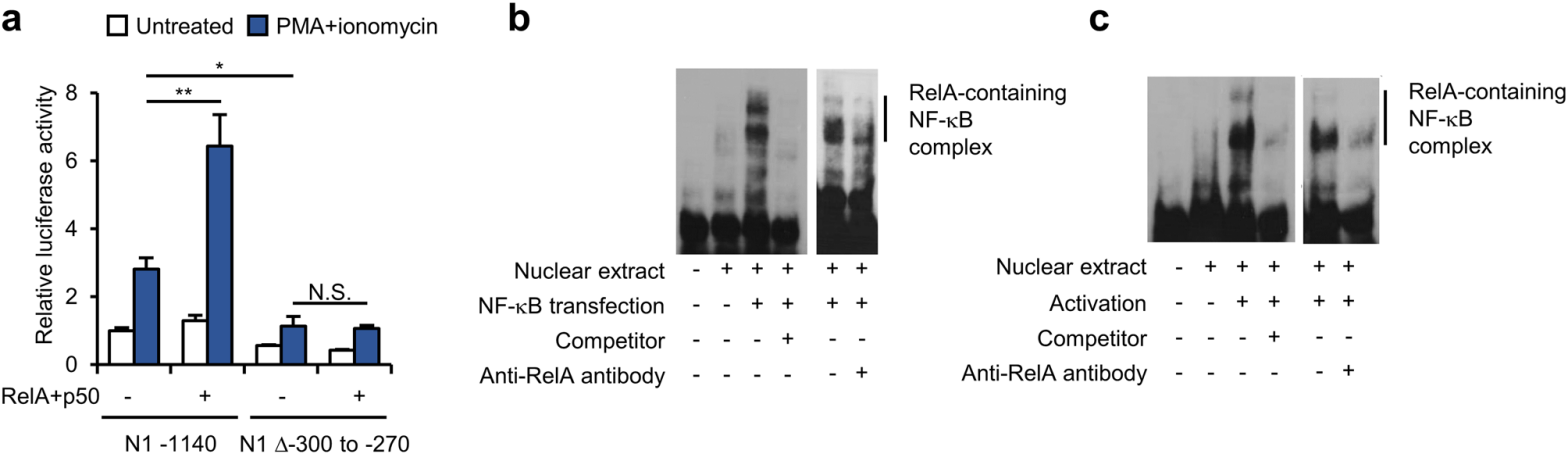
Validation of the predicted binding proteins for the *Notch1* − 300 to − 270 sequence. (**a**) Luciferase reporter assay performed in activated Jurkat T cells that were overexpressing RelA or p50. (**b, c**) An electrophoretic mobility shift assay was performed using a *Notch1* − 300 to − 270 sequence probe, and the probe-NF-κB complex was disrupted by an anti-RelA antibody in (**b**) RelA- and p50-overexpressing HEK293T cells, and (**c**) in activated Jurkat T cells. The original blots are presented in Supplementary Fig. 2. Data are presented as the mean ± SD. *P ≤ 0.05, **P ≤ 0.01, and ***P ≤ 0.001 (Student’s *t*-test). N.S., not significant.

To identify the transcriptional regulators that directly interact with the identified promoter region, in silico analyses of the *Notch1* − 300 to − 270 sequence were conducted using MatInspector and Transfac (Table 1 and Supplementary table 1). Transfac predicted SP1 to be the most likely molecule to bind to this region (Supplementary table 1). However, co-transfection of the SP1 expression vector with N1 − 1140 or N1 Δ − 300 to − 270 did not alter luciferase activity significantly (Supplementary Fig. 2). The analysis using MatInspector predicted that the NF-κB subunits p50 and RelA would be binding partners (Table 1). Interestingly, overexpression of p50 and RelA increased the transcriptional activity induced by the N1 − 1140 construct, but deletion of the *Notch1* − 300 to − 270 region abolished this transcriptional activation (Fig. 3a). An electrophoretic mobility shift assay (EMSA) was then conducted to confirm that these RelA-containing NF-κB subunits bind to the *Notch1* − 300 to − 270 sequence via specific sequences. Nuclear extracts of p50- and RelA-overexpressing HEK293T cells interacted with the *Notch1* − 300 to − 270 sequence probe (Fig. 3b), and addition of an anti-RelA antibody disrupted formation of the probe-NF-κB complex (Fig. 3b). In addition, we assessed binding of the RelA-containing NF-κB subunits to the *Notch1* − 300 to − 270 sequence in the Jurkat T cell system, and found that nuclear extracts from activated Jurkat T cells bound to the *Notch1* − 300 to − 270 sequence probe; in addition, formation of the probe-NF-κB complex was disrupted by an anti-RelA antibody (Fig. 3c). These data imply that sequence-specific direct binding of RelA-containing NF-κB proteins to the *Notch1* − 300 to − 270 region occurs in activated T cells.

**Table 1:** In silico prediction of RelA and p50 as binding proteins for the *Notch1* − 300 to − 270 sequence.

Detailed family information	Detailed matrix information	Tissue	Opt	Core sim	Matrix sim	Sequence
Nuclear factor κB/c-rel	NF-κB p50	Blood cells, Immune system, Leukocytes, Myeloid cells, Phagocytes	0.83	0.75	0.709	gggCGGAgcgcccgc
Nuclear factor κB/c-rel	NF-κB p65	Blood cells, Immune system, Leukocytes, Myeloid cells, Phagocytes	0.87	0.75	0.75	ggcgggcgCTCCgcc

Name	Sequence					
*Notch1* − 300 to − 270	$\frac{{{\text{p}}50}}{{\frac{{5^{\prime } {\text{CATAGGGGCGGAGCGCCCGCCC}}}}{{{\text{RelA}}}}}}$					
NF-κB binding motif	5′-GGGRNYY YCC-3′					

Opt, represents optimized threshold; Core sim, core similarity; Matrix sim, matrix similarity. Nucleotides in capital letters denote the core sequence by MatInspector.

### NF-κB upregulates Notch1 transcription in activated T cells

**Figure 4:**
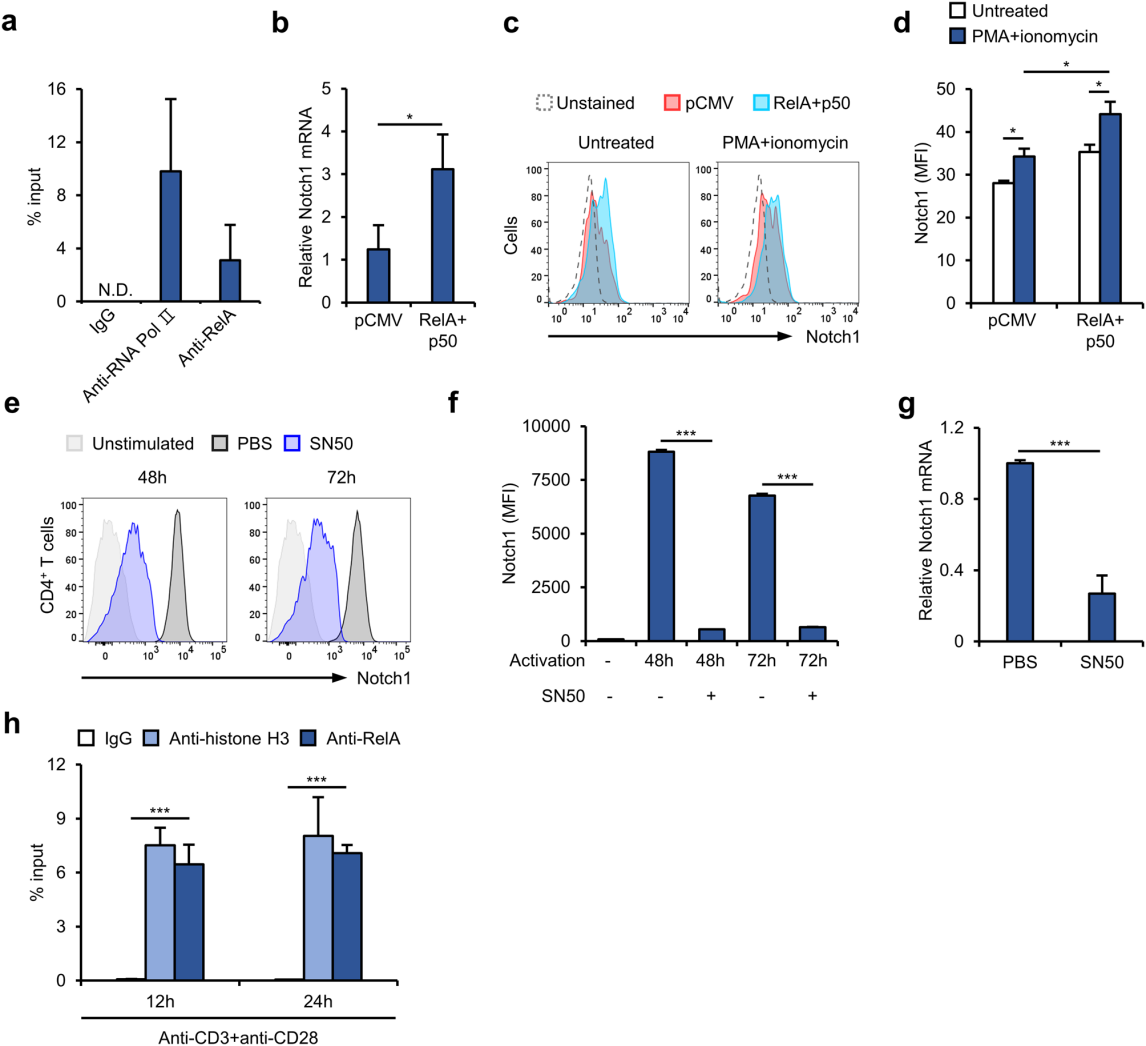
Upregulation of *Notch1* transcription by NF-κB in activated T cells. (**a**) Jurkat T cells activated with PMA/ionomycin were subjected to ChIP-qPCR analysis to detect chromatin containing the *Notch1* -300–-270 region. N.D., not determined. (**b**) Quantitative real-time PCR for *Notch1* mRNA expression in RelA and p50-overexpressing Jurkat T cells. (**c, d**) Flow cytometry for surface Notch1 expression in activated Jurkat T cells overexpressing RelA or p50. (**e**, **f**) Flow cytometry analysis of primary CD4^+^ T cells activated in the presence of 100 μM SN50 or PBS for the indicated times. (**g**) Quantitative real-time PCR of *Notch1* mRNA from activated primary CD4^+^ T cells cultured for 48 h in the presence of 100 μM SN50 or PBS. (**h**) ChIP-qPCR analysis of primary CD4^+^ T cells, activated with anti-CD3 and anti-CD28 antibodies for the indicated times, to detect chromatin containing the *Notch1* − 300 to − 270 region. Data are presented as the mean ± SD. *P ≤ 0.05, **P ≤ 0.01, and ***P ≤ 0.001 (Student’s *t*-test).

The physiological relevance of the interaction of NF-κB with the *Notch1* − 300 to − 270 region was next evaluated using a chromatin immunoprecipitation (ChIP) assay. Crosslinked and sheared chromatin fragments obtained from activated Jurkat T cells were precipitated using anti-RelA antibody, and then quantitative real-time PCR analysis was used to assess the activity of the *Notch1* − 300 to − 270 region using specific primers (Fig. 4a). This showed that NF-κB binds to the chromatin structure of the *Notch1* − 300 to − 270 region in activated T cells, and this binding occurred in both living cells and under molecular experimental conditions. Moreover, overexpression of RelA and p50 increased *Notch1* mRNA expression in Jurkat T cells (Fig. 4b); in addition, surface expression of Notch1 was increased by overexpression of these proteins (Fig. 4c, d). To further investigate the underlying mechanism(s), we exposed primary CD4^+^ T cells to SN50, an inhibitor of NF-κB activation, during CD3/CD28-mediated activation. Surface expression of Notch1 by vehicle-treated cells increased markedly after activation; however, this was abolished by treatment with SN50 (Fig. 4e, f). In addition, SN50 significantly decreased *Notch1* transcription in activated primary CD4^+^ T cells (Fig. 4g). Furthermore, a ChIP assay using activated primary CD4^+^ T cells revealed that chromatin fragments containing the *Notch1* − 300 to − 270 region were precipitated by an anti-RelA antibody, suggesting that *Notch1* expression in activated primary CD4^+^ T cells is regulated by the same mechanism (Fig. 4h). Taken together, these observations suggest that NF-κB directly binds to the *Notch1* − 300 to − 270 region and positively regulates the transcription of *Notch1*, leading to higher surface expression of Notch1.

## Discussion

The importance of Notch signaling during T cell development has been well defined. Critically, deletion or inactivation of Notch1 causes development of the B cell lineage, rather than the T cell lineage, in mouse model systems^11,12^. In addition, Notch1 is upregulated in CD4^+^ T cells upon stimulation using anti-CD3 and anti-CD28 antibodies^22^. We have shown that the surface expression and mRNA expression of Notch1 are highly dependent on the CD28-PI3K pathway in activated T cells. PDK1 is regarded as the principal downstream effector of PI3K in CD4^+^ T cells, which implies that Notch1 plays a role in the PI3K-PDK1 downstream signaling complex and has roles in CD4^+^ T cell activation via PI3K-PDK1 downstream signaling cascades. Several studies of the roles of Notch1 in mature T cells have shown that Notch1 positively regulates expression of cytokines, interferon-γ, and interleukin-10^22,23^. However, conflicting data have been obtained regarding the roles of Notch1 during CD4^+^ T cell activation and proliferation. Some previous studies of the roles of Notch1 in CD4^+^ T cell activation and proliferation show stimulatory effects^23–25^, whereas others show inhibitory effects^9,26^. Therefore, a more detailed assessment regarding the exact roles of Notch1 in mature CD4^+^ T cell activation is required.

NF-κB participates in multiple processes in CD4^+^ T cells, including in their development, activation, survival, and differentiation^27^. The present findings suggest that NF-κB binds directly to the *Notch1* promoter and upregulates its transcription during cellular activation. This is important because stimulation of the TCR/CD28 complex is fundamental for most T cell activities, and because NF-κB is one of the principal downstream effectors of the CD28-PI3K-PDK1 signaling pathway^28,29^. For example, CD4-specific deletion of *Pdk1* impairs IκBα degradation under anti-CD3 and anti-CD28 simulation conditions^29^, and a recent study shows that PDK1-mediated NF-κB activation is essential for maintenance of regulatory T cells^30^. We showed that the inhibition of NF-κB activation by SN50 robustly disrupts *Notch1* transcription during primary CD4^+^ T cell activation, although issues such as the possible effects of SN50-mediated inhibition of AP-1 and NFAT^31^ on Notch1 expression remain unaddressed. Furthermore, we confirmed binding of NF-κB to the *Notch1* promoter region. In addition, Notch1 regulates NF-κB activity. Inactivation of Notch1 inhibits sustained NF-κB activity in T cells, and nuclear localization of NF-κB is regulated by the Notch1 intracellular domain^32,33^. Therefore, the interaction between Notch1 and NF-κB may represent a feedback mechanism whereby the activity or expression of each molecule is maintained, and might drive T cell activation or other downstream processes. However, it is unclear whether such feedback regulation would have positive or negative effects on T cell activities, because the roles of Notch1 in CD4^+^ T cell activation and proliferation are as yet poorly defined. Furthermore, the results of another study suggest an opposing effect of Notch1 on NF-κB activity^34^.

Activating mutations of Notch1 are regarded as a key diagnostic target for T-ALL, because uncontrolled activation of Notch1 signaling could be oncogenic^35^. In addition, the constitutive activation of NF-κB in T-ALL cells^36^ and higher expression of *Notch1* in patients with T-ALL^37^ have been reported. As discussed above and as indicated by the present and previous findings, feedback regulation between Notch1 and NF-κB may occur, including in T-ALL cells. Interruption of this feedback regulation through targeting of the interaction of Notch1 with NF-κB may therefore represent a means of reducing *Notch1* expression. The therapies for T-ALL have improved in recent years, and these include several inhibitors that target molecules that are essential for Notch1 signaling, such as γ‑secretase inhibitors, ADAM inhibitors, SERCA inhibitors, and monoclonal antibodies that target Notch1^38^. However, their use might be associated with problems, including toxicity and limited efficacy, and a complete cure is difficult to achieve in some patients because of relapse or resistance to the therapy. Therefore, more diverse approaches to the regulation of Notch1 activity should be developed, such as transcriptional or epigenetic approaches. For example, modulation of the transcription of *Notch1* might have synergistic effects with those of other drugs. However, most previous studies regarding *Notch1* transcriptional regulation did not use a T cell system, and suggested a broad range for the *Notch1* promoter sequence. Therefore, a deeper understanding of the regulatory mechanisms in T cells may provide clues regarding alternative therapeutic approaches for T-ALL.

We have shown that the expression of Notch1 is greatly increased during T cell activation, and that the *Notch1* − 300 to − 270 region is critical for the positive regulation of *Notch1* transcription in activated T cells. Moreover, NF-κB proteins, which are generally regarded as being downstream of PDK1, have been identified as transcriptional activators that directly bind to the *Notch1* − 300 to − 270 region. Thus, upregulation of Notch1 is mediated via the PI3K-PDK1-NF-κB signaling pathway in activated T cells. Furthermore, the present findings were not made solely in a lymphoblast cell line: similar findings were made in primary CD4^+^ T cells isolated from mice. A better understanding of the detailed mechanisms of the transcriptional regulation of *Notch1* in T cells may provide opportunities for further progress in research into autoimmune diseases and immunomodulatory therapies.

## Methods

### Vectors and mice

The *Notch1* start codon (+ 1) to − 1140 region was amplified from mouse genomic DNA by PCR and subcloned into the pGL3 basic (N1 − 1140 vector). The N1 − 800, − 600, − 400, − 350, − 300, − 250, − 200, and − 150 vectors were constructed by truncating the N1 − 1140 vector by PCR and subcloning into the pGL3 basic vector. The forward primers used for constructing the vectors were as follows: N1 − 1140: 5′-GGGACGCGTGAGATAAAGCATGAGAGGCT-3′; N1 − 800: 5′-TTCACGCGTAGCCCTAGGGACAGG-3′; N1 − 600: 5′-TCCACGCGTATGTCGCAGAGGGCG-3′; N1 − 400, 5′-TTCACGCGTCCACCAAAGCGCTGG-3′; N1 − 350, 5′-TTCACGGCTGGAACCAGGGGCGGA-3′; N1 − 300, 5′-TTCACGCGTCATAGGGGCGGAG-3′; N1 − 250, 5′-TTCACGCGTCGGGAGGGAGCGCA-3′; N1 − 200 5′-TTCACGCGTGTGCGAGCGCAGTGA-3′; N1 − 150, 5′-TTCACGCGTCGCTGAGAGCCCAGC-3′; and 5′-TTGAAGCTTGCCTGCTCGCCAGCTGCC-3′ was used as the common reverse primer. The N1 Δ − 300 to − 270 and N1 Δ − 280 to − 250 vectors were constructed by deletion of these regions from the N1 − 1140 vector. pRenilla, pcDNA3-p50, pCMV-RelA, and pMigR1 vectors were used for transfection.

*Pdk1*^*flox/flox*^* Cd4-Cre* mice were generated as previously described^29^. The mice were held in a specific pathogen-free facility at Gwangju Institute of Science and Technology. Eight-to-ten-week-old wild-type C57BL/6 mice, *Pdk1*^*flox/flox*^* Cd4-Cre*, and *Pdk1*^+*/flox*^* Cd4-Cre* mice were euthanized by CO_2_ inhalation prior to use in the experiments. All animal experiments were approved by the Seoul National University’s Institutional Animal Care and Use Committee (SNU-210315-7-5) and were performed in accordance with relevant guidelines and regulations. The study was carried out in compliance with the ARRIVE guidelines.

### Cell culture and flow cytometry

HEK293T cells were cultured in DMEM supplemented with 10% FBS and Jurkat cells, and mouse CD4^+^ T cells were cultured in RPMI-1640 supplemented with 10% FBS. Jurkat cells were activated by adding 500 ng/ml PMA and 100 ng/ml ionomycin to the culture medium, or anti-human CD3 and anti-human CD28 antibodies. Mouse CD4^+^ T cells were isolated from the spleens and lymph nodes of C57BL/6 mice using a negative isolation kit (Stem Cell Technologies, Vancouver, Canada). To activate the mouse CD4^+^ T cells, they were cultured in plates to which anti-mouse CD3 and anti-mouse CD28 antibodies were bound. Inhibitors were used at the following concentrations: 50 μM for LY294002 and 100 μM for SN50. Antigen-presenting cells were sorted from mouse mononuclear cells and then cocultured with mouse CD4^+^ T cells. Cell lines were transfected with FuGENE HD (Promega, Madison, WI, USA) or Lipofectamine 2000 (Thermo Fisher Scientific, Waltham, MA, USA), according to the manufacturer’s protocol. The electroporation of mouse CD4^+^ T cells was performed using a Nucleofector kit for mouse T cells (Lonza, Basel, Switzerland).

Jurkat cells were co-transfected with pMigR1 to select transfected cells, which were sorted according to their GFP expression. Mouse cells were mixed with monoclonal antibodies purchased from eBioscience: CD3ε-PerCP/Cy5.5, CD4-eFlour 450, TCRβ-FITC, and Notch1-APC. These antibodies were used at a 1:200 dilution in PBS containing 2% serum. The cells were acquired using a FACSAria III sorter (BD, Franklin Lakes, NJ, USA) or a Guava EasyCyte analyzer (Merck Millipore), and the data were analyzed using FlowJo software (BD).

### Nuclear fractionation

Cells were harvested and collected in tubes, and the cell pellets were washed, resuspended in a hypotonic buffer (10 mM HEPES pH 7.9, 10 mM KCl, 0.1 mM EDTA, 1 mM DTT, 0.5 mM PMSF, and 1 μg/ml leupeptin), and then incubated on ice for 15 min. NP-40 was added to 0.05%, and the mixture was vigorously vortexed. The supernatant was removed after centrifugation at 1000 × *g* for 5 min at 4 °C, and the remaining pellet was washed with hypotonic buffer, resuspended in a hypertonic buffer (20 mM HEPES pH 7.9, 400 mM NaCl, 1 mM EDTA, 1 mM DTT, 1 mM PMSF, and 1 μg/ml leupeptin), and incubated on ice for 30 min, with vigorous vortexing. Nuclear extracts were obtained by centrifugation at 20,000 × *g* for 10 min at 4 °C.

### In silico analysis

Conserved sections of the *Notch1* promoter region were searched for using Genome Browser (https://genome.ucsc.edu/). MatInspector (https://www.genomatix.de/matinspector/) and Transfac (https://genexplain.com/transfac/) were used to identify transcription factors that might bind to the *Notch1* − 300 to − 270 region.

### Quantitative real-time PCR

RNA was isolated from Jurkat or mouse CD4^+^ T cells using a RNeasy mini kit (Qiagen, Hilden, Germany). Up to 1 μg of RNA was converted to cDNA by reverse transcription (Enzynomics, Daejeon, Korea). SYBR green 2 × premix (Enzynomics) was used for quantitative real-time PCR on a CFX Connect real-time PCR detection system (Bio-Rad Laboratories, Hercules, CA, USA). Ct values were calculated for each target gene using a standard curve, and expression is stated relative to that of *Gapdh*.

### Dual luciferase reporter assay

Ten thousand cells per well were seeded onto a 24-well plate and transfected with a reporter plasmid mixture (composed of pGL3 basic-based *Notch1* vector and pRenilla) and other overexpression vectors. The cells were activated using PMA/ionomycin or anti-CD3 and anti-CD28 antibodies. They were harvested by centrifugation at 500 × *g* for 5 min at 4 °C and then washed twice using PBS. The luciferase activity of the samples was measured using the Dual Luciferase Reporter Assay System (Promega) according to the manufacturer’s protocol.

### Electrophoretic mobility shift assay

Nuclear extracts of NF-κB-transfected HEK293Ts and activated Jurkat cells were prepared as described above. The *Notch1* − 300 to − 270 sequence was synthesized as a biotinylated probe (Macrogen, Seoul, Korea), and then the nuclear extracts and biotinylated probes were incubated together at room temperature. An anti-RelA antibody (Santa Cruz Biotechnology, Dallas, TX, USA) was added to the mixture to disrupt formation of the probe-NF-κB complex. The mixtures were then separated on a 6% native polyacrylamide gel and transferred to a nylon membrane, which was incubated at 80 °C to permit crosslinking. The biotinylated DNA probes were then detected using a LightShift® Chemiluminescent EMSA Kit (Thermo Fisher Scientific) and exposure to X-ray films.

### Chromatin immunoprecipitation assay

Crosslinked chromatin was extracted from 2 × 10^7^ Jurkat cells or 5 × 10^6^ mouse primary CD4^+^ T cells and sonicated to shear the DNA, and then sheared crosslinked chromatin DNA was incubated with protein G agarose slurry for 1 h at 4 °C, with gentle rotation. After removing the agarose by centrifugation, the precleared supernatant was incubated with 5 μg anti-RelA antibody overnight at 4 °C, with gentle rotation. Positive control samples were incubated with 1 μg of anti-RNA polymerase II antibody or anti-histone H3 antibody, and negative control samples were incubated with 1 μg of normal mouse IgG. Protein G agarose was then added to each sample, which was incubated for 1 h at 4 °C with gentle rotation. Protein G agarose was washed and put through elution. In addition, the purified DNA was used for quantitative real-time PCR analysis. ChIP was performed using an EZ-ChIP kit (Merck, Darmstadt, Germany), according to the manufacturer’s protocol.

## Statistical analysis

The significance of differences between two groups was analysed using Student’s t-test. All data are expressed as the mean ± SD. For all statistical tests, *P*-values ≤ 0.05 were considered significant.

## Supplementary Information

Supplementary Information.

## Data Availability

All data generated or analysed during this study are included in this published article (and its Supplementary Information files).
