# Supplementary material for: Transcriptional regulation of Notch1 by nuclear factor-κB during T cell activation
Source: Sci Rep. 2023 Jan 2;13:43. doi: 10.1038/s41598-022-26674-1 (PMC9807580; doi:10.1038/s41598-022-26674-1)
Supplement: Supplementary file 1 — Supplementary Information. [file 41598_2022_26674_MOESM1_ESM.docx]

**Supplementary information**

**Supplementary figure 1. Evaluation of Notch1 mRNA stability after LY294002 treatment.** Quantitative PCR analysis of mouse primary CD4^+^ T cells activated with anti-CD3 and anti-CD28 antibodies in the presence of 50 μM LY294002 or DMSO (vehicle), followed by treatment with 10 μg/ml actinomycin D. (a) Schematic representation of the experimental flow. (b) Kinetics of Notch1 mRNA decay. Error bars represent mean ± SD.


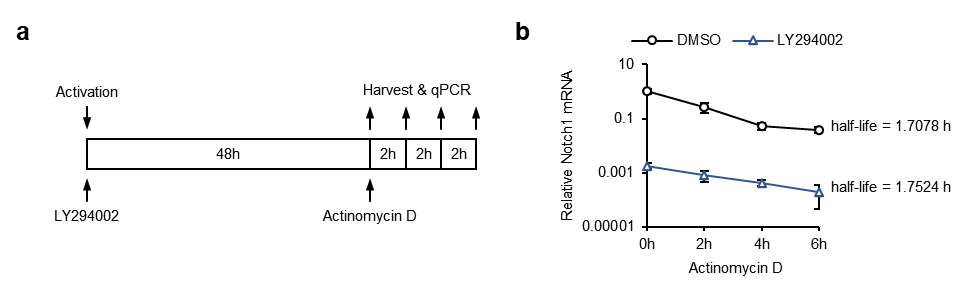


**Supplementary figure 2. Assessment of the role of SP1 during regulation of Notch1 promoter activity.** Luciferase reporter assay of Jurkat T cells under the indicated conditions. Data are presented as the mean ± SD.

**
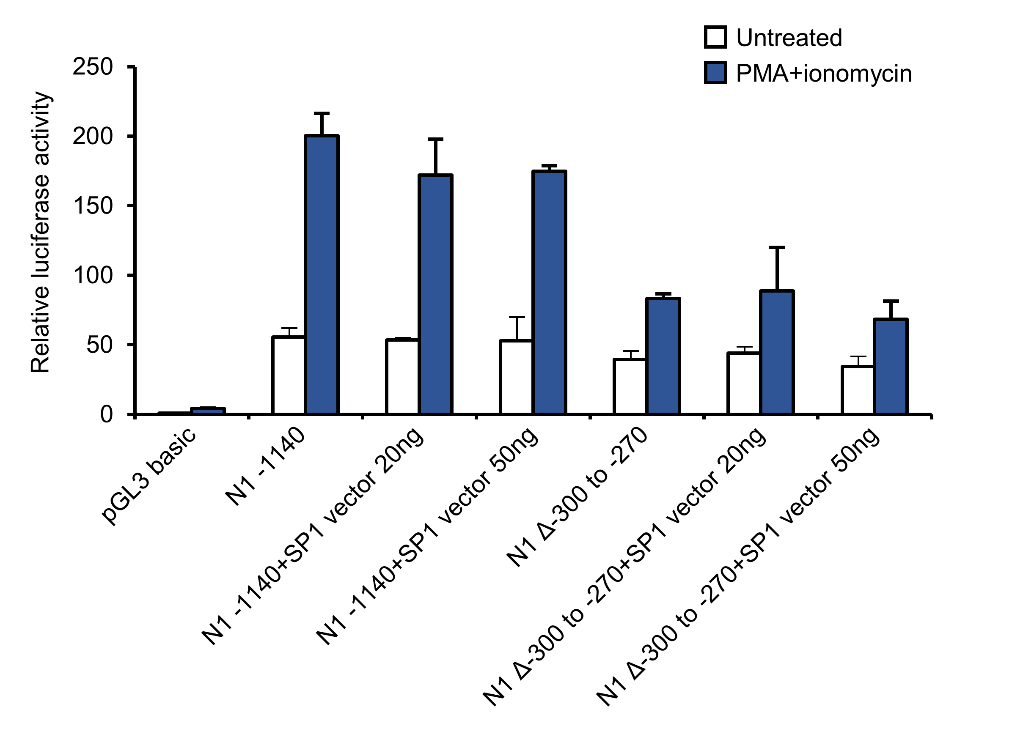
**

**Supplementary figure 3. Original image of DNA electrophoresis.** Original image of the gel in Figure 2b.


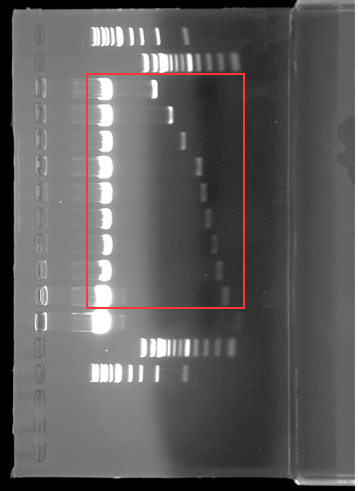


**Supplementary figure 4. Original images for the electrophoretic mobility shift assay.** (a–d) Original images of the data in (a) Figure 3b, left panel; (b) Figure 3b, right panel; (c) Figure 3c, left panel; and (d) Figure 3c, right panel.


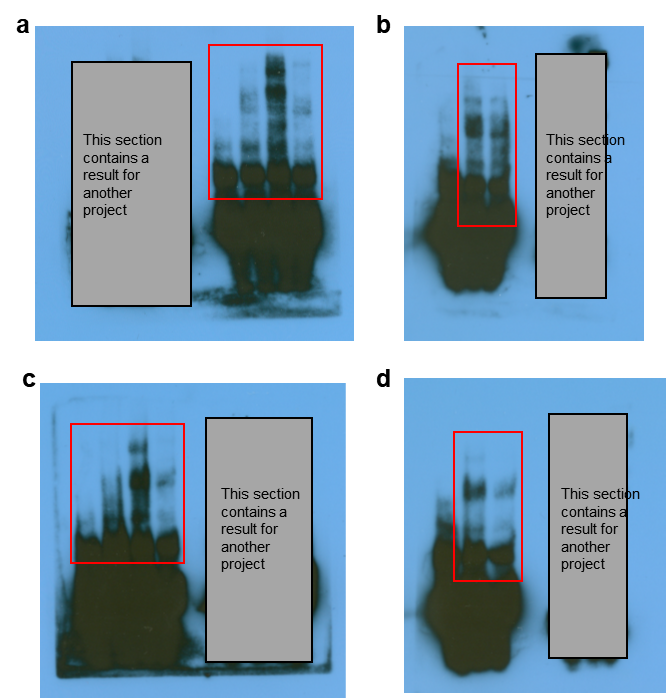


**Supplementary table 1. Notch1 −300 to −270 sequence binding proteins predicted by Transfac.**

| Name | Position | Core match | Matrix match | Sequence |
| --- | --- | --- | --- | --- |
| Stimulating protein 1 (SP1) | 1+ | 1 | 0.974 | catag**GGGC**ggagcgcc |
| E2F transcription factor (4E2F4) | 3+ | 1 | 0.91 | taggg**GCGG**agcgcccg |
| Myeloid zinc finger protein1 (MZF1) | 3+ | 1 | 0.894 | ta**GGGG**cggag |
| Transcription factor II B (TFIIB) | 15+ | 1 | 0.881 | gcc**CGCC** |
| Transcription factor II B (TFIIB) | 11+ | 1 | 0.877 | gag**CGCC** |
| E2F transcription factor 2 (E2F2) | 8+ | 1 | 0.8 | gcgga**GCGC**ccgcccgc |
| NME/NM23 nucleoside diphosphate kinase1 and 2 | 3+ | 1 | 0.791 | ta**GGGG**cggagcgcccg |
| Kruppel-like factor 3 (KLF3) | 4+ | 1 | 0.782 | aggggc**GGAG**cgcccgccc |
| Glial cells missing homolog 1 | 11+ | 1 | 0.715 | gagcg**CCCG**cccgct |
| Glial cells missing homolog 1 | 15+ | 1 | 0.715 | gcccg**CCCG**ctcaga |
